# Pain Management, Functional Recovery, and Stress Response Expressed by NLR and PLR after the iPACK Block Combined with Adductor Canal Block for Total Knee Arthroplasty—A Prospective, Randomised, Double-Blinded Clinical Trial

**DOI:** 10.3390/jcm12227088

**Published:** 2023-11-14

**Authors:** Malgorzata Domagalska, Tomasz Reysner, Grzegorz Kowalski, Przemysław Daroszewski, Aleksander Mularski, Katarzyna Wieczorowska-Tobis

**Affiliations:** 1 Department of Palliative Medicine, University of Medical Sciences, 61-701 Poznań, Poland; 2 Department of Spine Disorders and Pediatric Orthopedics, Poznan University of Medical Sciences, 61-545 Poznań, Poland; 3 Department of Forensic Medicine, Institute of Medical Sciences Collegium Medicum, University of Zielona Góra, 65-046 Zielona Góra, Poland

**Keywords:** pain management, neutrophil-to-lymphocyte ratio, platelet-to-lymphocyte ratio, stress response, peripheral nerve block, regional anaesthesia, iPACK, adductor canal block

## Abstract

Introduction: This study aimed to investigate pain management, functional recovery, and stress response expressed by the neutrophile-to-lymphocyte ratio (NLR) and platelet-to-lymphocyte ratio (PLR) after the popliteal artery and posterior knee capsule infiltration (iPACK) block combined with adductor canal block (ACB) in total knee arthroplasty (TKA). Patients and Methods: This was a prospective, double-blinded, randomised, controlled trial in a tertiary referral hospital. Three hundred and sixty-six patients were randomly allocated into the sham block group and iPACK combined with the ACB group. The primary outcome was postoperative pain scores. The secondary outcomes were opioid consumption, functional recovery expressed by a range of motion, and quadriceps strength. Also, the neutrophil-to-lymphocyte ratio (NLR) and platelet-to-lymphocyte ratio (PLR) were calculated. Results: There were significant differences between the sham block and iPACK + ACB group in pain scores *p* < 0.0001 at all time points. Therefore, there was a significant difference in opioid consumption (*p* < 0.0001) and functional recovery (*p* < 0.0001). Also, NLR and PLR levels 12 h (*p* < 0.0001) and 24 h (24 h) after surgery (*p* < 0.0001) were much lower in the iPACK + ACB group. Conclusion: After total knee arthroplasty, the iPACK combined with ACB block group improved pain management, functional recovery, and stress response. Therefore, we strongly recommend this technique as a part of a multimodal analgesia protocol in knee surgery.

## 1. Introduction

Total knee arthroplasty (TKA) is one of the most frequent and most painful procedures in orthopaedic surgery, and extreme pain immediately after surgery has been reported in half of TKA patients [1]. Optimal pain control is crucial for patient satisfaction and comfort, enhancing patient mobilisation, and hospital discharge [2]. Multimodal analgesia has been assimilated into most clinical lanes to improve patient comfort, satisfaction, and ambulation [3]. Different analgesic strategies, including motor-sparing local infiltration analgesia (LIA) and peripheral nerve blocks, lower pain scores, and opioid consumption, thus support early functional recovery [4,5,6].

Adductor canal block (ACB) is an alternative to femoral nerve block (FNB) after TKA. ACB provides analgesia to intra-articular and anteromedial parts of the knee. In contrast to FNB, ACB preserves quadriceps muscle strength, thus facilitating functional recovery [7]. However, posterior knee pain is often present after TKA. The ultrasound-guided infiltration between the popliteal artery and the capsule of the posterior knee (iPACK) block was established to avoid this obstacle. The iPACK block was designed to hit the posterior terminal sensory nerve branches [8]. Despite two meta-analyses concerning the iPACK and ACB in TKA [9,10], there is no unity regarding whether the iPACK added to ACB reduces pain scores and opioid consumption and promotes functional recovery.

The neuroendocrine hormones and cytokines are released during surgery and anaesthesia. Also, postoperative pain during TKA represents inflammatory, nociceptive, and neuropathic pain and matches surgical stress response. Leukocytosis, lymphopenia, and neutrophilia develop in return for surgery. The neutrophil-to-lymphocyte ratio (NLR) and platelet-to-lymphocyte ratio (PLR) are extensively used in many branches of medicine as prompt markers of immune replay to different noninfectious stimuli [11,12]. Recently, it has been proved that regional anaesthesia reduces stress response expressed by NLR and PLR [13,14,15]. However, to date, no studies have been conducted to assess the outcomes of peripheral nerve block on NLR and PLR in knee surgery.

This study intended to evaluate the accoutrements of iPACK and ACB on TKA by comparing postoperative pain, opioid consumption, functional recovery, and NLR and PLR levels between the sham block and iPACK combined with ACB. Therefore, this is the first trial that investigates the effects of peripheral nerve block on NLR and PLR in patients undergoing TKA. 

## 2. Patients and Methods

### 2.1. Study Design and Participants

The prospect of this trial was conducted by the Declaration of Helsinki at the Independent Public Health Care Institution of the Ministry of the Interior and Administration in Poznan, Poland. The Institutional Review Board of the Poznan University of Medical Sciences approved the study policy on 17 June 2020, protocol 495/20, and it was registered with clinicaltrails.gov (NCT06086483). Written informed consent was collected from all patients for this research program.

Enrolment was introduced before surgery for adults undergoing elective primary unilateral total knee arthroplasty under spinal anaesthesia, American Society of Anesthesiologists physical status 1, 2, or 3, and aged >18 years.

Patients were not admitted to this study if they denied participating, had an adverse reaction to any of the drugs used in the study, had an infection of the site of needle puncture, had a history of opioid abuse, had ASA > 3, were less than 18 years of age, had known or suspected coagulopathy, had a liver failure or renal failure (predicted glomerular filtration rate of <15 mL/min/1.73 m^2^), had pre-existing anatomical or neurological disorders in the lower limbs, or had severe psychiatric illness or intellectual problems in pain evaluation. Also, patients with blood transfusions during the perioperative period and whose estimated blood loss during surgery was over 30% of the total blood volume were excluded from the study.

### 2.2. Randomisation

Patients were randomly assigned to receive ultrasound-guided iPACK blocks with ACB blocks or sham blocks using a computer software-generated 1:1 randomised list using the nQuery Advisor program (Statistical Solutions, Boston, MA, USA). The randomisation list was given to an impartial investigator who camouflaged group assignments in serially numbered, closed, blurred boxes. An expert specialist tracked management to disclose the envelopes before the nerve block administration to announce the group allotment and execute the procedure. The anaesthesia team, surgeons, operating room staff, and patients were unaware of the study group assignment. Group unblinding and uncovering appeared once the statistical investigation was finished.

### 2.3. Perioperative Management and Spinal Anaesthesia Procedure

All the patients underwent routine spinal anaesthetic management as commonly practised in our hospital. Patients in both groups were given 7.5 mg of midazolam orally and 8 mg of dexamethasone intravenously half an hour before the surgery as a part of pre-emptive multimodal analgesia. Then, 100 mg of fentanyl and 2 mg of midazolam for mild sedation were administered intravenously before induction of anaesthesia. Additionally, all patients underwent spinal anaesthesia while the patient was seated, and 20 mg of 0.5% ropivacaine was injected into the L3–L4 space with a 27G Whitacre needle. After spinal anaesthesia and before surgery, 1 g of tranexamic acid and 1 g of cefazolin were injected intravenously. No periarticular infiltration occurred by the surgeon during the surgery.

### 2.4. iPACK (Infiltration between the Popliteal Artery and the Capsule of the Posterior Knee) Block Procedure (Figure 1)

The transducer was placed transversely over the medial aspect of the knee, 2–3 cm above the patella. The transducer was slid proximally to identify the distal femoral shaft and popliteal artery. The needle was inserted in-plane, from the anteromedial facet of the knee, into the space between the femur and popliteal artery. When the posterior part of the popliteal artery was reached, 2 mL of the 0.5% ropivacaine was injected to confirm the proper needle position. Additionally, 20 mL of 0.5% ropivacaine was injected into the space between the popliteal artery and the posterior knee capsule.

**Figure 1:**
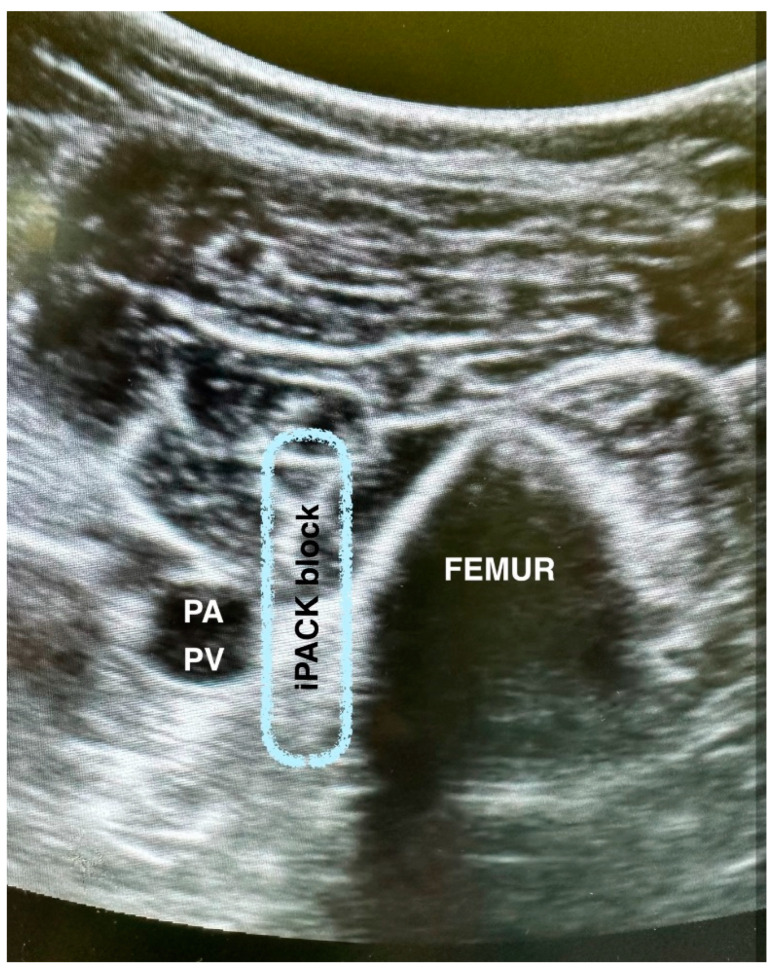
Infiltration between the popliteal artery and the capsule of the posterior knee (iPACK) block procedure. PA—popliteal artery; PV—popliteal vein.

### 2.5. Adductor Canal Block Procedure (Figure 2)

The transducer was located in a transverse orientation at the middle level of the middle third of the thigh. The femoral artery, sartorius muscle, and adductor longus muscle were identified. The needle was inserted in-plane in a lateral to a medial direction and advanced toward the femoral artery and saphenous nerve. After the negative aspiration, 1 mL of local anaesthetic was injected to confirm the proper injection site. A total of 10 mL of 0.5% ropivacaine was administered. 

**Figure 2:**
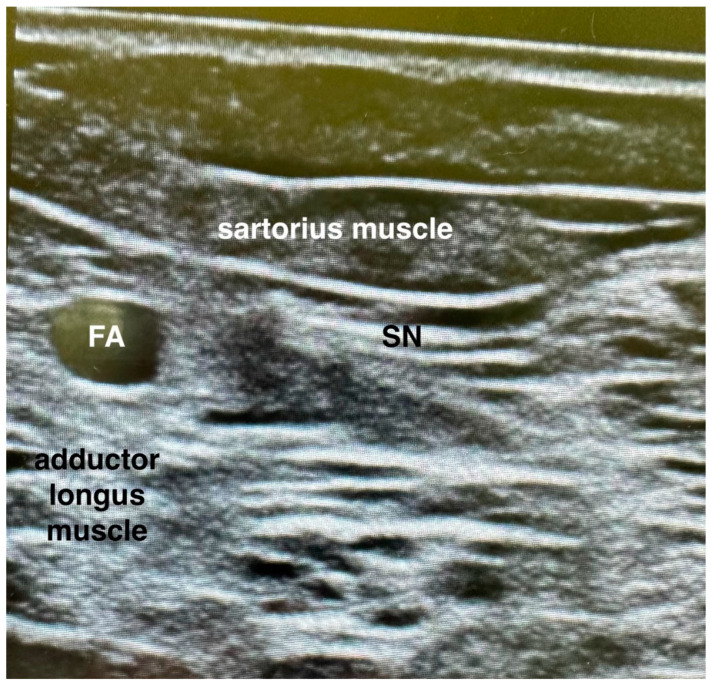
Adductor canal block procedure. FA—femoral artery; SN—saphenous nerve.

### 2.6. Surgical Techniques

All TKA procedures were performed by two surgical teams using a standard medial parapatellar approach without a tourniquet. Both measured gap-balancing and resection techniques were utilised. Cemented, fixed bearings and posterior-stabilised implants from the Stryker Triathlon knee system were implanted. One gram of intravenous tranexamic acid was given before surgery to reduce the blood loss.

### 2.7. Postoperative Analgesia Management and Evaluation of Outcomes

After the total knee arthroplasty ended, the patients were transmitted to the post-anaesthesia care unit (PACU). Postoperatively, a cold pack reduced pain for twelve hours after surgery. The same multimodal postoperative analgesia was applied in the PACU in both groups, which was consistent with ketorolac 50 mg every twelve hours. Also, metamizole 1 g i.v. and acetaminophen 1 g i.v. were both applied every six hours. If the NRS score was higher than 4, 5 mg morphine was administered as rescue analgesia. The 8 mg of ondansentrone was given when severe nausea or vomiting occurred. Enoxaparin for four weeks postoperatively was given as a thromboembolism prophylaxis. Finally, patients were ambulated with the help of the walker after the first ten postoperative hours.

### 2.8. Outcome Assessments

The primary outcome quota was pain scores at rest and during active flexion 45° to 5 days following surgery. At all postoperative time points (24, 48, 72, >72 h), patients were asked to rate perceived pain using an 11-point Numeric Pain Rating Scale (NRS: 0 indicating no pain and 10 indicating the worst pain imaginable) experienced at rest and during active flexion 45°. The secondary outcomes included time to first opioid use obtained from the postoperative and orthopaedic wards. Also, total opioid consumption was recorded 0–24, 24–48, and 48–72 h after surgery. The expenditure of the different types of postoperative opioid administration was converted to intravenous morphine equivalents according to Supplementary Table S1.

The range of motion and quadriceps strength was measured to grade the functional recovery of the knee joint after surgery. The protractor was used for the range of motion. The interval of activities was three times a day, 6 h apart, and we used the best value as the day’s value. The quadriceps strength was measured by asking the patient to flex the hip and knee and the complete knee extension. The outcome assessor used resistance to knee extension and palpated the thigh muscles to measure muscle strength. No muscle movement scored 0 points, no joint movement with muscle contraction scored 1 point, no gravity resistance with joint movement scored 2 points, gravity resistance scored 3 points, partial counterforce resistance with gravity resistance scored 4 points, and normal joint function scored 5 points. The resulting judgement was accompanied by a group of two clinicians (GK and TR) who were satisfied with the group allotment.

Also, blood samples for NRL and PLR were collected 12 and 24 h after surgery.

### 2.9. Statistical Analyses and Sample Size Calculations

We studied our primary hypothesis that the iPACK block with ACB improves postoperative analgesia to calculate the sample size. Based on the published data on total knee arthroplasty using the iPACK block with ACB [16], we estimated pain score density as a mean of 4 and SD of 5. We use a limited Gaussian distribution with a range of 0 to 10, SD of 5, and a standard of 2 for the iPACK + ACB group to model the alluvion. We simulated a sample of 162 patients in each group under these hypotheses and two-sided = 5%. We estimated 95% power to catch differences in pain between groups as small as relatively 1 with an overall sample size of 324 subjects. Statistical analysis was accomplished using GraphPad Prism 8 software.

We used the Kołomogorov–Smirnov normality test to evaluate the parametric distribution of numerical variables. The Mann–Whitney U or Student’s *t* test resolved the deviations between groups. The analysis of contingency was correlated with Fisher’s exact test. Categorical variables were compared with the Mann–Whitney U test. Values are given as the mean (standard deviation), the median (interquartile range), or the number of patients (proportion). The equity of inpatient and operation eccentricity between the randomised groups was resolved by judging the standardised difference, which was corrected as the alteration in means or ratios prorated by the pooled standard deviation. Individually measured variables were delayed using a linear mixed exemplary with the patient mark as a random effect and group, time, and group-by-time cooperation as fixed effects, balancing for volatiles of operation and patient characteristics (sex, age, body mass index, ASA physical status, surgery duration). An unstructured covariance structure was practised. The Bonferroni correction was imposed to adapt for multiple resemblances. All analyses were adjusted using GraphPad Prism 8 software (GraphPad Software Inc., San Diego, CA, USA). A *p*-value of <0.05 was studied as statistically significant.

## 3. Results

### 3.1. Patients and Operation Characteristics

**Figure 3:**
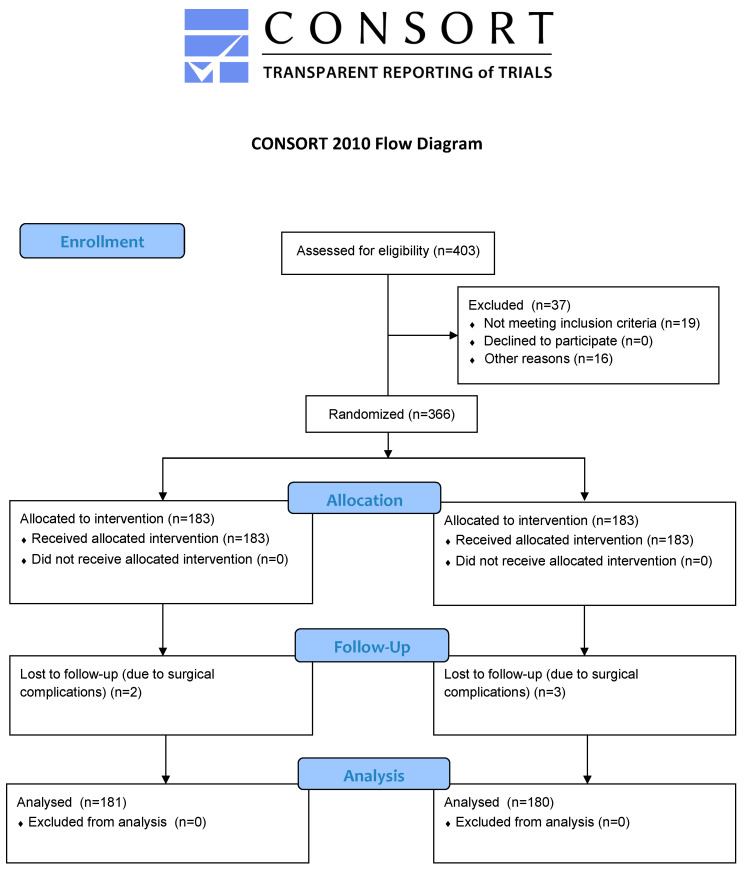
Consort flow chart.

Of 403 patients postponed for eligibility, 19 did not fit the inclusion criteria, and 16 chose general anaesthesia. The remaining 366 were randomly assigned to both groups. Another five were lost to follow-up as a result of surgical complications. Overall, 361 patients were inspected, as shown in Figure 3. No clinically admissible changes were possible from group characteristics, as shown in Table 1. 

### 3.2. Primary Outcomes

**Figure 4:**
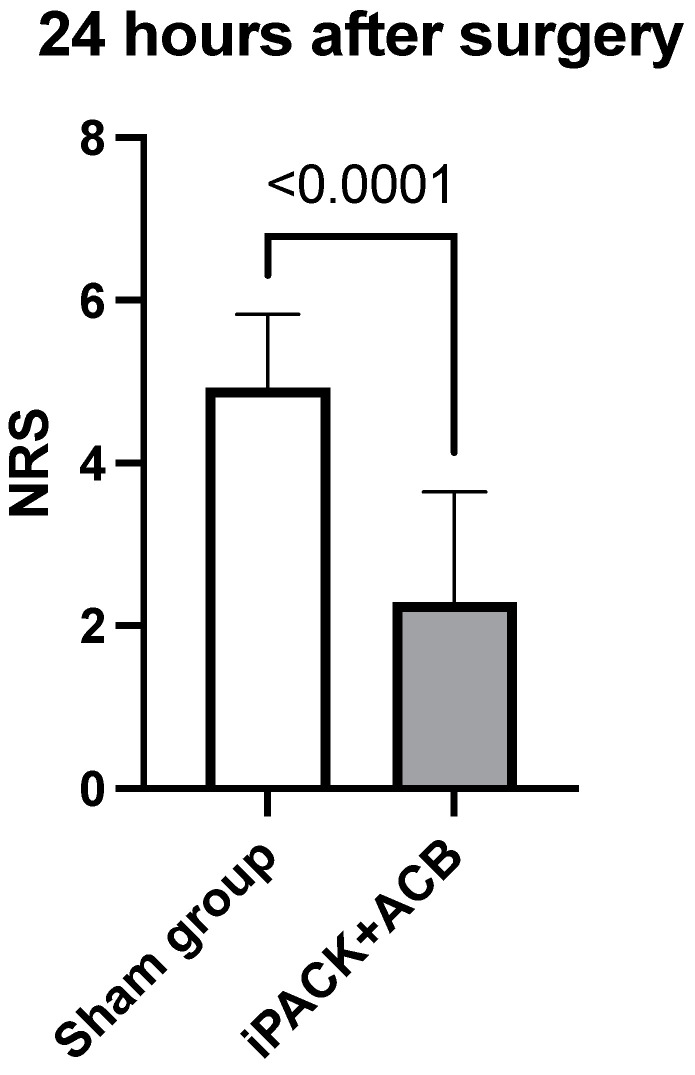
NRS 24 h. iPACK—a popliteal artery and posterior knee capsule infiltration; ACB—adductor canal block; NRS—Numeric Pain Rating Scale.

**Figure 5:**
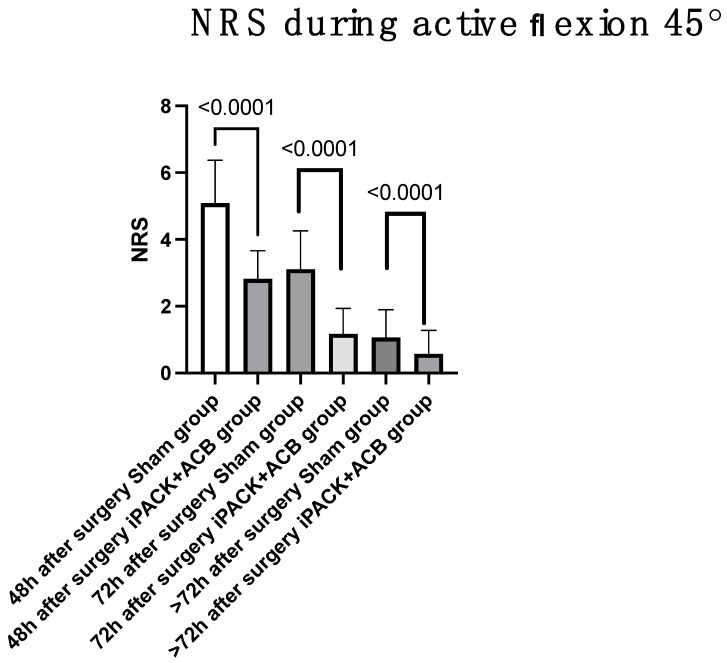
NRS during active flexion 45°. iPACK—a popliteal artery and posterior knee capsule infiltration; ACB—adductor canal block; NRS—Numeric Pain Rating Scale; h—hours.

Postoperative pain scores are demonstrated in Table 2 as well as Figure 4 and Figure 5. Patients who experienced the iPACK block with ACB had lower NRS pain scores at all time points. The iPACK block with ACB shows better pain control expressed by the NRS pain scores such as 4.9 vs. 2.3 at 24 h, *p* < 0.0001; 3.7 vs. 2.9 at 48 h, *p* < 0.0001; 2.0 vs. 1.2 at 72 h, *p* < 0.0001; 1.1 vs. 0.7 > 72 h, *p* = 0.0001, compared to the sham block.

Also, the NRS scores during active flexion 45° were decreased in the iPACK + ACB group at all time points. 5.1 vs. 2.9 at 48 h; 3.1 vs. 1.2 at 72 h; 1.1 vs. 0.6 >72 h, all *p* < 0.0001. 

### 3.3. Secondary Outcomes

Every patient in the sham group needed opioid pain medication for pain relief. In contrast, 74 (41%) in the iPACK + ACB group received none. 

**Figure 6:**
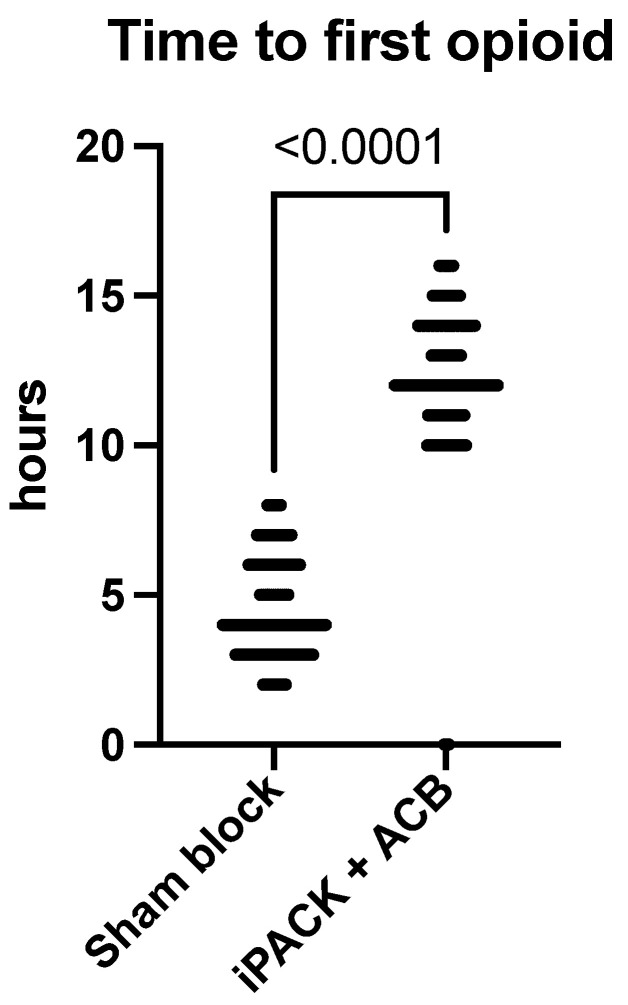
Time to the first opioid. iPACK—a popliteal artery and posterior knee capsule infiltration; ACB—adductor canal block.

Also, the time to first opioid was much longer in the iPACK + ACB group, 4.5 vs. 12.31 h, *p* < 0.0001 (Figure 6). 

In conclusion, the total opioid consumption deposited in intravenous morphine equivalents was decreased in the PENG group at all time points 10.2 vs. 1.7, *p* < 0.0001; 5.4 vs. 2.4, *p* < 0.0001; 0.7 vs. 0.4, *p* = 0.0123.

There was no difference in the quadriceps strength, 3.6 vs. 3.7, with *p* = 0.0857. However, the degree of knee range of motion was higher in the iPACK + ACB group, 87.4 vs. 85.2, *p* < 0.0001.

**Figure 7:**
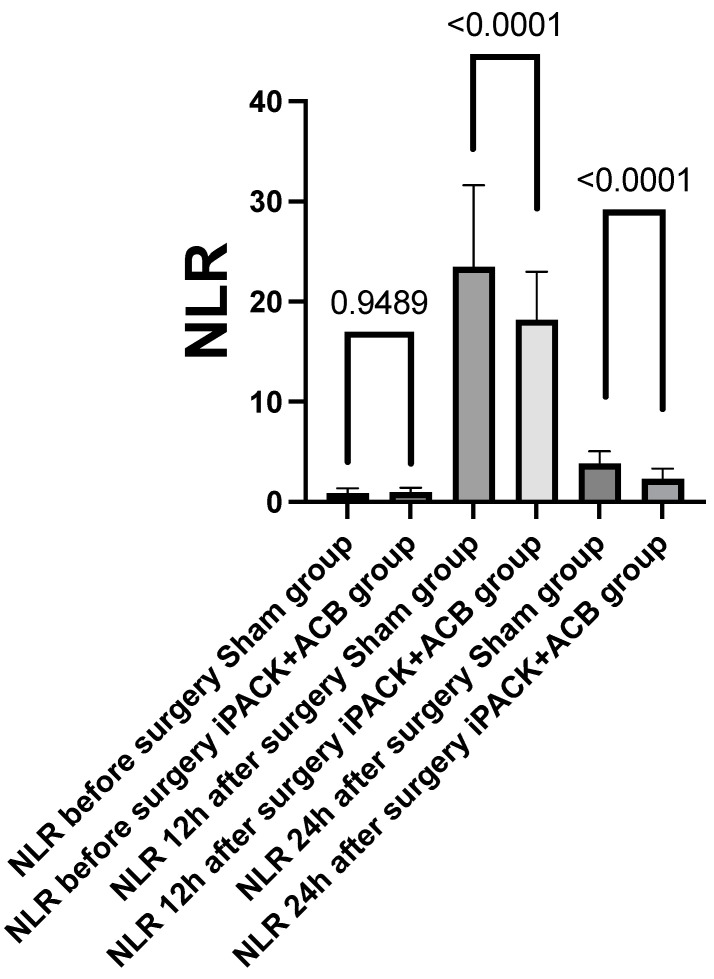
NLR—neutrophil to lymphocyte ratio; iPACK—a popliteal artery and posterior knee capsule infiltration; ACB—adductor canal block; NRS—Numeric Pain Rating Scale; h—hours;.

In addition, NLR levels were also lower in the iPACK + ACB group, 23.48 vs. 18.20 12 h after surgery and 3.84 vs. 2.31 24 h after surgery, both *p* < 0.0001 (Figure 7). 

Also, PLR levels were lower in the iPACK + ACB group, 531.6 vs. 2.08 24 h after surgery and 271.30 vs. 192.60 24 h after surgery.

The results are shown in Table 3.

## 4. Discussion

This prospective, double-blinded clinical trial showed that patients who received the iPACK block combined with ACB had better pain control, which was defined by the lower pain scores for over 72 h after surgery. Patients who received the sham block consumed significantly more opioids during hospitalisation than those who received the iPACK block combined with ACB. Also, patients who received two peripheral nerve blocks had better functional recovery, which was expressed by the range of motion and quadriceps strength. Additionally, patients with peripheral nerve blocks had lower stress response, which was defined by lower NLR and PLR levels 12 and 24 h after surgery.

Orthopaedic surgery involving the lower extremities, especially knee surgery, usually causes the most pain on the first day after surgery [1]. Inadequate pain control after TKA delays functional recovery and increases the rate of complications [17].

Sciatic and femoral nerve blockade is the standard for adequate analgesia after total knee arthroplasty. However, the motor block that develops after femoral and sciatic nerve blocks prevent early ambulation [6].

This impairment of muscle strength prompted the look for motor-sparing techniques.

We decided to perform ACB together with the iPACK block due to its motor-spraying effect and mechanism of action. ACB has been successfully used for pain management in knee surgery [18]. However, ACB only relieves pain in the peripatellar and intra-articular regions of the knee joint [19]; additional analgesia is then required to reduce pain [20].

Intraoperative periarticular injections (PAIs) are the standard analgesic option for pain management following TKA [21]. The drugs administered in PAI act much longer than surgery and thus contribute to pain management following TKA. PAI is performed by the orthopaedic surgeon using the landmark technic. Therefore, its effectiveness depends on the method and the analgesic regimen [22]. Also, PAI is associated with high volumes of local anaesthetic, which brings the risk of Local Anesthetic Systemic Toxicity (LAST). This limits the use of PAI in TKA.

The iPACK block is a relatively novel ultrasound-guided regional anaesthesia technique designed to block the small articular sensory branches of the popliteal plexus and the obturator nerve, resulting in analgesia of the posterior knee capsule. iPACK blockade targets the pain sensation below the knee and can relieve pain behind the knee without causing muscle weakness [9,23]. Therefore, the iPACK block, together with ACB, may be an effective tool for analgesia after TKA. Our results suggest that an iPACK block combined with ACB can provide sufficient analgesia after orthopaedic surgery with much lower opioid consumption levels than a sham block. Other trials also have shown similar conclusions that an iPACK block with ACB can achieve better motor-sparing pain management in the immediate postoperative period compared to standard pain management protocols [9,24,25].

The iPACK block with ACB has been documented to provide significant analgesia and early mobilisation following knee surgery due to its quadriceps strength-sparing effect [16,19,26]. However, most recent evidence is limited to trials with small group sizes [9,19]. We showed that iPACK with ACB not only decreases the pain sores and opioid consumption but also does not reduce the quadriceps strength and slightly improves ROM, which is similar to other studies [27,28,29].

Peripheral nerve block suppresses the formation of proinflammatory cytokines related to the stress response [30]. Also, peripheral nerve blocks provide an inflammatory and sympathetic response due to expanded blood flow, vascular permeability, and leukocyte aggregation. NLR and PLR are counted from platelet, neutrophil, and lymphocyte values from the complete blood count. They are inflammatory signals that anticipate morbidity, mortality, and subclinical inflammation [31].

Prosthetic joint infection (PJI) is one of the most severe complications after TKA [32]. Several studies have shown that PJI after knee surgery is associated with higher NRL and PLR levels [33,34,35,36,37]. Golge et al. [38] found the value of 2.45 to be a cut-off point for infection after TKA. We showed that the NLR levels after iPACK and ACB are much lower than in patients without peripheral nerve block. What is more important, we revealed that 24 h after surgery, the NLR values were lower than 2.45 in patients with the iPACK block combined with ACB compared with patients with sham block.

On the other hand, Tirumala et al. [36] revealed that PLR levels are strongly associated with PJI. He showed that values >234.13 are associated with a higher incidence of PJI. In our study, 24 h after surgery, PLR values were <200 in patients with the iPACK block combined with ACB compared to patients with the sham block.

Also, NLR levels correlate with venous thromboembolism (VTE), which is a severe complication after TKA [39]. Seo et al. [40] revealed that high NLR levels are a separate predictor of VTE in patients undergoing total knee replacement (TKR). In our study, NLR levels were much lower in the iPACK combined with ACB group than in the sham group.

To date, no studies have characterised the influence of peripheral nerve blocks on NLR and PLR levels following knee surgery. Our analysis indicates that iPACK with ACB reduces the surgical stress response expressed by NLR and PLR. Therefore, iPACK with ACB lowers the risk of PJI and VTE following TKA.

However, this study has limitations. The large volume of the local anaesthetic used for the iPACK block and the ACB is a single-shot injection rather than a catheter. We did not obtain the dermatome levels, the duration of the block, and the hospital discharge times. Also, we only calculated NLR and PLR 12 and 24 h after surgery.

## 5. Conclusions

Based on our findings, ultrasound-guided iPACK with ACB lowers the pain scores and reduces opioid consumption. Due to its motor-sparing mechanism of action, iPACK with ACB enhances functional recovery in patients undergoing TKA.

Also, iPACK combined with ACB lowers NRS and PLR levels, thus reducing the stress response.

Thus, we firmly advocate for this approach as part of a multimodal analgesia agreement in knee surgery.

## Figures and Tables

**Table 1:** Baseline characteristics. Values are mean (SD) or number.

	Sham Block *n* = 181	iPACK + ACB *n* = 180	*p* Value
ASA	2.5 (0.5)	2.6(1.6)	0.7469
Age (years)	68.5 (4.3)	67.8 (4.9)	0.3837
Sex (F/M)	54/126	50/130	0.7273
BMI (kg/m^2^)	31.4 (2.9)	31.1 (3.3)	0.3137
Surgery duration (min)	62.9 (7.4)	61.5 (6.7)	0.3253
Intraoperative blood loss (mL)	124.6 (40.8)	122.2 (40.0)	0.4745
Postoperative drainage volume (mL)	367.8 (44.4)	370.7 (40.3)	0.5361
NRS level before surgery Rest Flexion Ambulation	2.5 (0.5) 5.0 (0.7) 4.8 (0.8)	2.5 (0.5) 5.2 (0.8) 4.8 (0.8)	0.5635 0.0611 0.4708

iPACK—popliteal artery and posterior knee capsule infiltration; ACB—adductor canal block; ASA—American Society of Anesthesiologists; F—female; M—male; kg—kilograms; m—meter; min—minutes; ml—millilitres; NRS—Numeric Pain Rating Scale.

**Table 2:** Primary study outcomes. Values are mean (SD) or number.

	Sham Block Group *n* = 181	iPACK + ACB Group *n* = 180	*p* Value
**NRS postoperative**			
24 h	4.9 (0.9)	2.3 (1.4)	<0.0001
**NRS et rest**			
48 h	3.7 (0.8)	2.9 (0.9)	<0.0001
72 h	2.0 (0.7)	1.2 (0.8)	<0.0001
>72 h	1.1 (0.8)	0.7 (0.7)	0.0001
**NRS during active flexion 45°**			
48 h	5.1 (1.3)	2.9 (0.8)	<0.0001
72 h	3.1 (1.1)	1.2 (0.8)	<0.0001
>72 h	1.1 (0.8)	0.6 (0.7)	<0.0001

iPACK—a popliteal artery and posterior knee capsule infiltration; ACB—adductor canal block; NRS—Numeric Pain Rating Scale; h—hours.

**Table 3:** Secondary study outcomes. Values are mean (SD) or numbers.

	Sham Group *n* = 181	iPACK + ACB *n* = 180	*p* Value
**Postoperative opioid consumption**			
yes	181 (100%)	107 (59%)	<0.0001
no	0	74 (41%)	
**Time to first opioid**			
hours	4.5 (1.6)	12.31 (2.4)	<0.0001
**Total opioid consumption** **(Intravenous morphine equivalents; mg)**			
0–24 h	10.2 (2.6)	1.7 (1.6)	<0.0001
24–48 h	5.4 (1.2)	2.4 (2.5)	<0.0001
48–72 h	0.7 (1.1)	0.4 (0.8)	0.0123
**Functional recovery**			
Degree of knee range of motion	85.2 (4.7)	87.4 (4.4)	<0.0001
Quadriceps strength	3.6 (0.5)	3.7 (0.5)	0.0857
**NLR**			
Before surgery	1.80 (11.38)	0.97 (0.44)	0.9489
12 h	23.48 (8.13)	18.20 (4.79)	<0.0001
24 h	3.84 (1.24)	2.31 (1.04)	<0.0001
**PLR**			
Before surgery	187.9 (51.77)	180.10 (43.74)	0.1325
12 h	531.6 (131.1)	260.08 (4.84)	<0.0001
24 h	271.30 (57.22)	192.60 (52.68)	<0.0001

iPACK—popliteal artery and posterior knee capsule infiltration; ACB—adductor canal block; NRS—Numeric Pain Rating Scale; h—hours; NLR—neutrophile-to-lymphocyte ratio; PLR—platelet-to-lymphocyte ratio.

## Data Availability

The study datasets are available from the corresponding author upon reasonable request.

## Supplementary Materials

The following supporting information can be downloaded at: https://www.mdpi.com/article/10.3390/jcm12227088/s1, Table S1: the opioid used and the morphine equivalents.
