# Supplementary material for: Pain Management, Functional Recovery, and Stress Response Expressed by NLR and PLR after the iPACK Block Combined with Adductor Canal Block for Total Knee Arthroplasty—A Prospective, Randomised, Double-Blinded Clinical Trial
Source: J Clin Med. 2023 Nov 14;12(22):7088. doi: 10.3390/jcm12227088 (PMC10672046; doi:10.3390/jcm12227088)
Supplement: Supplementary file 1 [file jcm-12-07088-s001.zip › jcm-2711007-supplementary.pdf]

Supplementary Table S1. Supplementary table for the opioid used and the morphine equivalents.

	Equianalgesic dosing (mg)		*MME conversion factor*
	PO	*IV*	
**Morphine**	30	10	1
**Oxycodone**	20	/	1.5
**Nalbuphine**	/	8	0.8

MME – morphine milligram equivalent
